# Continuous theta-burst stimulation over the dorsolateral prefrontal cortex inhibits improvement on a working memory task

**DOI:** 10.1038/s41598-018-33187-3

**Published:** 2018-10-04

**Authors:** Teodóra Vékony, Viola Luca Németh, Adrienn Holczer, Krisztián Kocsis, Zsigmond Tamás Kincses, László Vécsei, Anita Must

**Affiliations:** 1 Department of Neurology, University of Szeged, Szeged, Hungary; 2 MTA-SZTE Neuroscience Research Group, Szeged, Hungary; 3 Institute of Psychology, University of Szeged, Szeged, Hungary

## Abstract

Theta-burst stimulation (TBS) over the dorsolateral prefrontal cortex (DLPFC) may be more effective for modulating cortical excitability compared to standard repetitive transcranial magnetic stimulation. However, the impact of intermittent (iTBS) and continuous TBS (cTBS) on working memory (WM) is poorly studied. The aim of our study was to compare the effects of iTBS and cTBS on WM over the left and right DLPFC. iTBS, cTBS or sham stimulation was administered over the right and left hemisphere of fifty-one healthy human subjects. WM was assessed before and after TBS using the 1-back, 2-back, and 3-back tasks. We found classical practice effects in the iTBS and the sham group: WM performance improved following stimulation as measured by the discriminability index. However, this effect could not be observed in the cTBS group. We did not find any hemisphere-dependent effects, suggesting that the practice effect is not lateralized, and TBS affects WM performance in a comparable manner if administered either over the left or the right hemisphere. We propose that our findings represent a useful addition to the literature of TBS-induced effects on WM. Moreover, these results indicate the possibility of clarifying processes underlying WM performance changes by using non-invasive brain stimulation.

## Introduction

Non-invasive brain stimulation has become a highly prosperous field of cognitive research in the last few decades. One of the available methods is repetitive transcranial magnetic stimulation (rTMS), that involve the application of rapid, brief magnetic fields to the scalp. These magnetic fields induce a current in the underlying tissues, leading to the depolarization of neurons in the targeted brain area^1^. Administering high-frequency rTMS (above 5 Hz) over the dorsolateral prefrontal cortex (DLPFC) has been shown to improve working memory (WM). In contrast, applying low-frequency (≤1 Hz) rTMS results in performance disruption^2–4^. A relatively new and seemingly more effective alternative to rTMS is theta-burst stimulation (TBS), which uses gamma frequency trains applied at theta rhythm^5^. The pattern of TBS was designed to mimic theta-gamma coupling, i.e. the modulation of gamma power by theta phase^6^. This type of cross-frequency coupling serves as a tool for realizing effective coordination between several cortical areas during cognitive tasks^7–9^. Thus, it might be capable of inducing behavioral effects as theta-gamma coupling is involved in WM and long-term memory processes^8,10–12^. Two main type of TBS can be distinguished: the intermittent TBS (iTBS) is described as facilitating^13^, while the continuous TBS (cTBS) is expected to be suppressive on cortical excitability^14^. However, in the motor domain, TBS after-effects have been found to be highly variable across subjects^15–18^. Despite the advantages of TBS (its specific pattern, shorter stimulation period and longer-lasting effects, at least in the motor domain)^5,19^, to date, only a few studies investigated the impact of TBS on WM^20^. Here we present a study comparing iTBS- and cTBS-induced effects on WM both over the right and left DLPFC.

Previous studies have found different effects of cTBS and iTBS on several cognitive domains. Interestingly, the distinct protocols did not consistently lead to opposite behavioral outcomes^20,21^. Three recent studies have investigated the effect of iTBS administration over the left DLPFC. Increased theta weighted phase-lag index between frontoparietal regions and increased parietal gamma power has been found following iTBS along with behavioral improvement in the n-back WM task^22^. Enhanced amplitudes of TMS-evoked event-related potentials^23^, as well as increased theta and gamma power, have also been detected by Chung *et al*.^24^. However, in these studies, consistent behavioral improvement could not be revealed^23,24^. On the other hand, cTBS over the DLPFC has been reported to impair WM^25,26^ and also to decrease theta power^27^. Given these inconsistencies, a systematic investigation of cTBS- and iTBS-related impact on WM and direct comparisons of the effect of the two stimulation protocols with the same stimulation parameters on WM are needed.

A recent study has compared changes in performance on several neurocognitive tests, both following iTBS and cTBS. WM performance decreased following both active stimulation protocols considering reaction times, as reflected by a diminished practice effect. However, only cTBS had a disruptive effect on task accuracy^21^. This suggests that cTBS does not (only) disrupt WM *itself* but (also) the capacity to improve in a WM task. Although practice effects have been considered to be a confounding factor in cognitive measurements, practice-related changes - as a tool to measure cognitive plasticity^28^ – also yield useful information about the underlying mechanism of cognitive performance^29,30^. It is currently not known whether the mechanism behind the practice-related improvement (i.e. the ability to learn task-specific skills) in a WM task can be interfered by a single session of non-invasive stimulation. This question might also be relevant for the interpretation of TBS effects on brain plasticity, which is a key mechanism for learning and memory^16,18^.

The above-mentioned TBS-studies have applied stimulation over the left DLPFC only. Thus, effects of TBS administration over the right hemisphere also remain an open question. A recent study has found that left but not right cTBS induces bilateral blood oxygenation changes in the prefrontal area^31^, and dopamine release is enhanced only following left DLPFC stimulation^32^. From the behavioral perspective, a direct comparison of TBS effects on WM over the left and right DLPFC is lacking in the current literature. Traditional TMS studies have found that verbal WM appears to be modified by left but not right DLPFC stimulation^33,34^. Others have reported rTMS to effectively modulate WM both over the left and the right DLPFC^3,35–38^. However, in some cases, the effects of rTMS over the right DLPFC have only been prominent for WM tasks containing spatial^39,40^ or negatively valenced stimuli^41,42^. Thus, clarifying whether DLPFC stimulation over the left and the right hemisphere is associated with verbal WM changes is warranted.

The above-mentioned gaps in the literature have raised the following questions for our study: (1) is iTBS improving and cTBS worsening WM performance, (2) are left and right DLPFC stimulation leading to different consequences considering their distinct effect on WM performance? Therefore, we administered iTBS, cTBS or sham stimulation over the left and right DLPFC of healthy participants. The n-back task, a well-established approach for WM assessment, has been administered twice: once before and once after the stimulation. Hoy *et al*. found behavioral improvement on WM tasks, as well as increased fronto-parietal theta synchronization and parietal gamma band power following iTBS^22^. Therefore, we expected an increase in performance on the n-back task following iTBS administration. Opposite behavioral and electrophysiological effects using cTBS have been reported by Schicktanz *et al*.^25^ and Chung *et al*.^24^, respectively. Thus, we hypothesized that cTBS would decrease WM performance. Our third hypothesis was that the effects related to TBS would not be equal over the two hemispheres. Based on previous findings, we expect more pronounced results following left DLPFC stimulation compared to right DLPFC stimulation^31,33,34,43^.

## Results

### Overview

A total of fifty-one participants completed the study. Subjects received iTBS, cTBS or sham stimulation over the DLPFC. At two separate occasions, the left and right hemisphere were stimulated, respectively. The n-back working memory task (1-back, 2-back, and 3-back) was completed by the participants both prior to and following the stimulation. Performance was assessed by reaction times and d′ scores.

### Pre-stimulation RTs and d’ scores

We did not find pre-stimulation differences between groups regarding both median RTs and d′ scores. This is supported by non-significant GROUP main effects and the lack of interactions including GROUP (all p > 0.05) (for details, see Supplementary Tables S1 and S2). These results indicate an equal pre-stimulation performance of the three groups for all administered levels of the n-back task and for each condition.

### Median RTs

The mixed ANOVA for median RTs revealed a significant main effect of TIME (F_(1,48)_ = 8.763, p = 0.005, n_p_^2^ = 0.154). Post-hoc tests showed slightly shorter overall median RTs following stimulation (mean scores: pre-stimulation 0.618 s ± 0.012 SE; post-stimulation 0.600 s ± 0.011 SE). An additional main effect of LOAD was found (F_(2,96)_ = 115.233, p < 0.001, n_p_^2^ = 0.706), indicating increasing RTs with higher cognitive demand (mean scores: 0.499 s ± 0.009 SE for 1-back; 0.609 s ± 0.014 SE for 2-back; 0.719 s ± 0.017 SE for 3-back). The interaction of the two main effects was also found to be significant (F_(1,96)_ = 4.947, p = 0.009, n_p_^2^ = 0.093). Post-hoc comparison revealed that the difference between the two time points was detectable for the 2-back (F_(1,48)_ = 9.137, p = 0.004, n_p_^2^ = 0.160) and 3-back level (F_(1,48)_ = 6.519 p = 0.014, n_p_^2^ = 0.120), but not for 1-back (F_(1,48)_ = 0.591, p = 0.446, n_p_^2^ = 0.012, BF_10_ = 0.200). The above interaction was not modified by GROUP (F_(4,96)_ = 1.358, p = 0.254, n_p_^2^ = 0.054), or by SIDE (F_(1.689,81.095)_ = 0.314, p = 0.694, n_p_^2^ = 0.007). To clarify whether there is truly no difference related to stimulation type, or our data are just not sensitive enough to detect the difference, we calculated Bayes-factor excluding (H_0_) and including (H_1_) the effect of GROUP. The Bayes-factor model indicated a strong evidence for H_0_ (BF_10_ = 0.050). We applied the similar algorithm for SIDE, indicating strong evidence in favor of H_0_ (BF_10_ = 0.064).

### d’ scores

**Figure 1:**
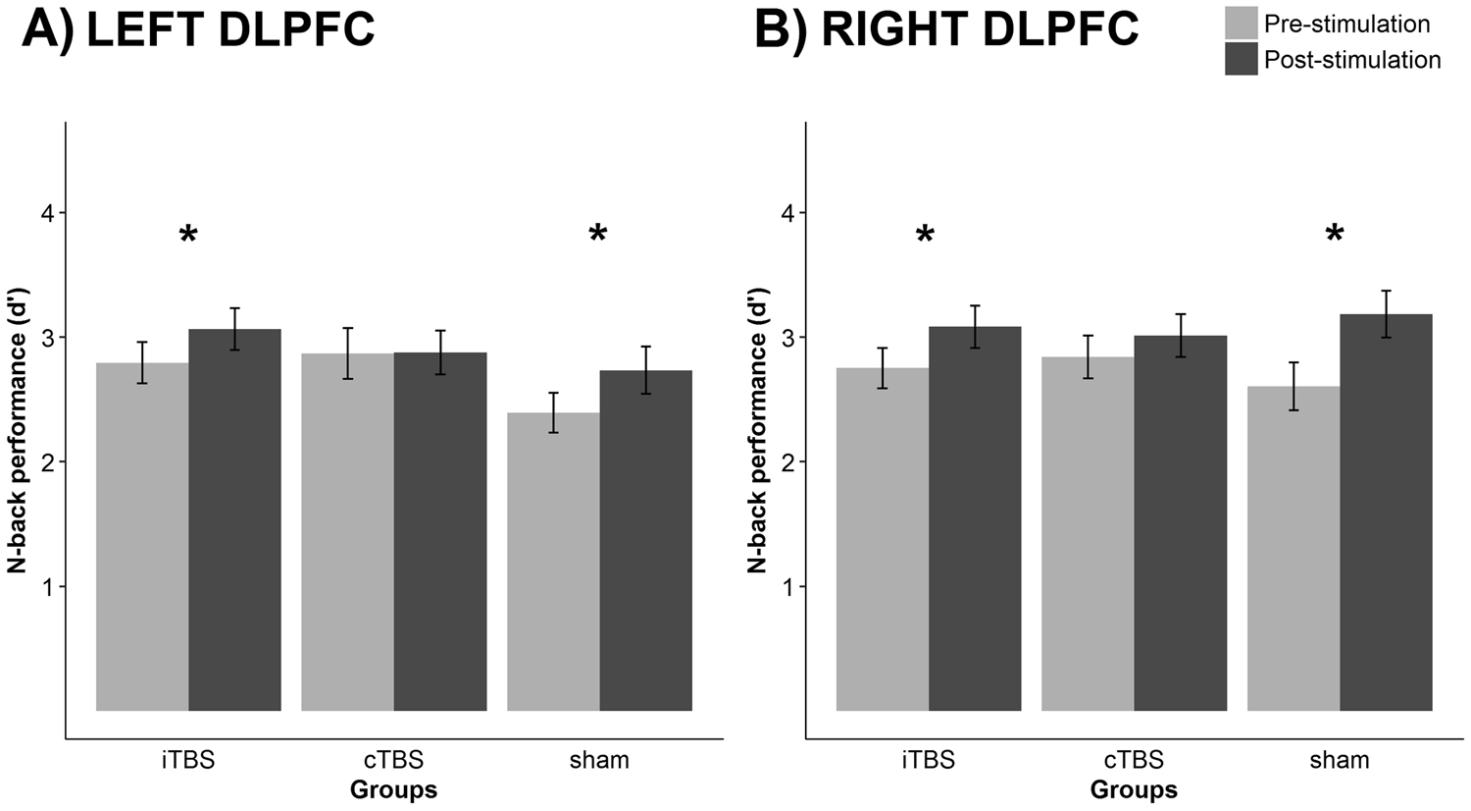
Pre- and post-stimulation n-back performance (d′ scores on 2-back and 3-back) when stimulating over the left (**A**) and right (**B**) DLPFC. Error bars indicate the standard error of mean. d′ scores improved in the iTBS and sham, but not in the cTBS group following stimulation.

The ANOVA of d′ scores revealed a significant main effect of LOAD (F_(1,48)_ = 344.822, p < 0.001, n_p_^2^ = 0.878), as well as TIME (F_(1,48)_ = 30.297, p < 0.001, n_p_^2^ = 0.387). Post-hoc tests showed better performance on the 2-back compared to 3-back condition, indicating a higher cognitive demand for the 3-back condition (mean scores: 3.575 ± 0.065 SE for 2-back; 2.125 ± 0.088 SE for 3-back). As for the main effect of TIME, increased performance was found following stimulation, signaling a potential practice effect between the two time points (mean scores: 2.708 ± 0.072 SE for pre-stimulation; 2.992 ± 0.071 SE for post-stimulation). Notably, a significant TIME × GROUP interaction was detected (F_(2,48)_ = 4.252, p = 0.02, n_p_^2^ = 0.151). To further analyze the source of this interaction, pairwise comparison with estimated marginal means was applied. We found a difference between the two time points in the iTBS (F_(1,48)_ = 12.095, p = 0.001, n_p_^2^ = 0.201) and the sham group (F_(1,47)_ = 25.113, p < 0.001, n_p_^2^ = 0.343), but this did not apply for the cTBS group (F_(1,48)_ = 0.999, p = 0.323, n_p_^2^ = 0.02, BF_10_ = 0.398) (Fig. 1). The interaction above was not modified by SIDE (F_(1,48)_ = 0.198, p = 0.821, n_p_^2^ = 0.008). To support the finding that SIDE as a factor has no critical effect on the detected interaction, Bayes-factors were calculated excluding (H_0_) and including (H_1_) for the effect of SIDE. In this model, Bayes-factor indicated strong evidence in favor of H_0_ (BF_10_ = 0.096).

## Discussion

In the current study, we aimed to investigate the effects of iTBS and cTBS delivered over the right and left DLPFC on two repeated measurements of WM performance. The n-back task has been administered before and after stimulation. The expected practice effect (i.e. general improvement due to repeated task completion^44^) occurred in the iTBS and sham stimulation group. This was reflected by the discriminability index. However, cTBS eliminated the practice effect and thus reducing WM performance. Considering RTs, practice-related effects occurred independently of stimulation type. Moreover, we detected a similar impact of TBS on both hemispheres. Bayesian statistics further support our findings obtained with more classical statistical approaches.

TBS has been shown to modulate brain activity by mimicking theta-gamma coupling^5^ which is a key feature for realizing coordination during cognitive tasks^7–9^. Thus, the effect of TBS is expected to modulate WM based on the theta-gamma neural code^11^. Previous studies have found a disruptive effect of cTBS on WM measured by behavioral methods^25,26^. Therefore, our results suggesting reduced WM performance (i.e. lack of improvement) following cTBS are in line with previous findings. We might argue that cTBS affected WM performance by causing long-term depression-like effects or by impairing the dopaminergic transmission in networks involving the DLPFC^5,32,45^. Besides that, the modulation of theta-gamma oscillations could also play a role in the cTBS-related WM effects. Studies have found cTBS to increase the power of theta-gamma frequency oscillations over the DLPFC, whereas iTBS has been reported to increase it^22,23,27^.

Contrary to our hypothesis, a lack of iTBS effects was found in the current study. iTBS has been reported to enhance task-related theta-gamma synchronization between frontal and parietal areas^22,24^, and subtle effects of iTBS on WM have also been shown^22^. However, some recent studies have also failed to find behavioral enhancement after iTBS accompanied by oscillatory changes^23,24^. Behavioral modulations are difficult to show after a single session of stimulation of healthy individuals^2^, and the behavioral consequences of cTBS on cognition might be more stable than iTBS effects^46^. This assumption could partially explain why we only detected cTBS-related changes on the behavioral level. Studies show that iTBS is more likely to alter electrophysiological markers (i.e. to cause differences in the features of event-related potentials, to change the degree of theta-gamma coupling or power in the crucial frequency ranges) than to cause behavioral changes^23,24^. Thus, iTBS might have had an effect on the neural level, which was not manifested on the behavioral level. Nevertheless, as WM-related electrophysiological after-effects of cTBS are not revealed yet, further studies should be conducted to clarify the underlying mechanism of the cTBS-induced behavioral changes, also compared to iTBS.

We might also speculate that the revealed disruptive effect of cTBS was not necessarily specific to WM *itself*, but rather to the consolidation of task-specific skills. Therefore, practice effects might also be interpreted as task-related knowledge which would help participants to improve their performance on the second administration. This might be supported by the lack of practice effect reported in neurocognitive disorders^47,48^, which are characterized by deficits in memory consolidation and acquiring new information^49^. Considering that memory processes may be modified by TBS^50,51^, and theta frequency oscillations are associated with memory consolidation processes^12^, it might be possible that prefrontal stimulation affected the consolidation of task-related skills. Future studies assessing whether consolidation may also play a role in TBS-related WM performance changes, seem warranted.

We did not find practice effects to occur in the 1-back condition. This supports the assumption that the changes detected here are WM-dependent to some extent and not solely associated with a more general task-related skill learning ability. Practice-related effects have been previously described to be independent of task difficulty^52^. However, different levels of performance modulation (or the lack of practice-related behavioral changes) following sham TBS have been found on several neuropsychological tasks^21^. A recent study has also revealed that the practice effect consists of various sub-processes, which are differently affected by the repeated completion of the task^53^. In the current study, processing speed was not modulated by TBS, thus supporting the notion that the applied stimulation affected only sub-processes defining WM performance. Nevertheless, the question if the measured effects originate from alterations in task-independent model-building processes or from solely WM-related changes remains in the focus of interest for future research. This could be addressed by measuring TBS-induced changes on the practice effect using other executive function tests with less WM demand (for example, Attention Network Task^54^). Additionally, the longitudinal assessment of practice effects (i.e. through multiple testing sessions) could reveal its dynamics and potential specificities. To sum up, we suggest that the disruption of practice effects related to cTBS might serve as a helpful concept to explain the inhibitory influence of cTBS on cognitive performance, whereas iTBS appears to be less robust than cTBS in this aspect.

Previous rTMS findings have reported bilateral contribution or even right DLPFC superiority for WM tasks using different cognitive targets^3,35–37,39,40,42^. In contrast, an early TMS study has suggested the left DLPFC stimulation leads to verbal WM performance modulation^33^. Interestingly, neuroimaging data have also suggested left DLPFC superiority for WM^43^. The n-back task used here was verbally featured, with no spatial or emotional aspects, which could partially explain the equal effects of TBS over the left and right hemisphere. We might argue that the left and right DLPFC play a similar role in the tested WM-related learning ability. Another possible explanation might be that disrupting activity in either hemisphere provokes an imbalance between the hemispheres that results in impaired performance^55^. This would also mean that enhancing activity using iTBS or high-frequency rTMS on one hemisphere could cause an imbalance that ultimately reduces performance. In studies using facilitatory TMS-methods either increase or a lack of change in performance has been detected^2,46^. This makes the hemispheric imbalance explanation less plausible. However, future studies specifically addressing this issue are required. Our current results further support the notion that WM-related processes can be equally modulated by TBS over either hemisphere in healthy individuals.

The stimulation intensity is crucial for the interpretation of our results considering that it differed remarkably from the intensity levels applied by previous studies testing the effects of TBS on WM. Nevertheless, cognitive changes have been described previously with comparable or even lower intensities^56–58^. The average MT of our participants was approximately 60% of the MSO. Thus, the stimulation intensity level was at nearly 50% of MT, compared to 80% of MT typically used in similar studies^22,25^. Consequently, we might speculate that cTBS exerts its effect on WM at low intensity, whereas iTBS might require higher intensities to have a more prominent or longer-lasting effect on WM performance. Additionally, iTBS and cTBS are characterized by distinct electrophysiological parameters, with the ideal stimulation intensity also differing if applied over motor cortical areas^5^. Similar to our findings, a diminished practice effect on n-back performance following cTBS has been previously found when comparing it to iTBS and sham stimulation^21^. The stimulation intensity applied by Viejo-Sobera *et al*.^21^ was 80% of the active motor threshold. This value is lower compared to motor threshold measured without voluntary contraction, typically by 5–20% of the MSO^59^. Thus, the level of intensity was comparable to our administration but lower than in studies reporting an increase in WM performance following iTBS^22^. Additionally, a recent study aimed to identify the ideal stimulation intensity for iTBS to modulate WM. This revealed an inverse U-shaped pattern of the different stimulation intensity effects, with nearly 75% of resting MT resulting in the largest neurophysiological changes. Nevertheless, 50% of resting MT also influenced neurophysiological functioning - but not at the behavioral level^24^. Exact data on the ideally recommended stimulation intensity of cTBS to affect WM is not yet available, therefore, future studies should address this issue. We strongly believe that our current results shed further light on potentially distinct but optimal stimulation intensities of different TBS protocols. This is supported by the disruptive effect of cTBS at the applied intensity, whereas iTBS remained ineffective at the same intensity.

In the present study, we found cTBS to have an inhibitory influence on the practice effect during the n-back task, while iTBS did not exert this fundamental effect. Our findings suggest that (1) the two distinct stimulation protocols presumably exert different effects on measured cognitive abilities, and (2) stimulation of the left or right DLFPC might have equivalent effects on WM at the behavioral level. Furthermore, in the light of previously reported neurophysiological changes related to WM following TBS administration^22,24,25^, future studies should examine post-TBS neuronal responses if stimulating both hemispheres. The role of practice underlying WM performance alterations and its interaction with TBS should be systematically explored in the future. The current results also emphasize the importance of investigating effects of low-intensity TBS on cognitive function and the potential differences of the ideal stimulation intensity for iTBS and cTBS to modulate WM. Above this, the assumption that iTBS and cTBS are defined by distinct ideal stimulation intensity levels could well influence the design of TBS-based treatment protocols. We strongly believe that the current findings represent a useful addition to the process of developing effective ways to reveal the influence of iTBS and cTBS on WM and to clarify the sub-processes behind WM changes by using non-invasive brain stimulation.

## Methods

### Participants

The selected sample size was based on an a priori sample size estimation. We calculated the required sample size with a medium estimated effect size, as TBS-induced effect sizes were found to be larger than in rTMS studies^5,22^. To find group differences between the three groups and within two repeated measurements with a power of 0.85, and assuming a medium effect size of 0.5, the required sample size was 48. Fifty-two healthy volunteers were recruited to participate in the study (see Table 1 for demographic data). None of the subjects had a history of any psychiatric or neurological disorder at the time of the participation. All participants had normal or corrected-to-normal vision. Participants were randomly assigned to one of the three groups and were naïve to the stimulation type. Informed consent was signed by all participants prior to the first session, and none of them withdrew from the experiment because of TBS discomfort. We had to exclude one participant due to administration failure in the first session. Thus, final analysis was carried out including fifty-one participants. The study was conducted in accordance with the Declaration of Helsinki, and the experimental protocol was approved by the Regional Scientific and Research Ethics Committee, Albert Szent-Györgyi Health Center, University of Szeged (Ref. no.: 165/2014).

**Table 1:** Demographical data.

	cTBS	iTBS	sham
Gender (f/m)	6/11	8/10	12/4
Mean age	24.23 ± 2.81 S.D.	25.27 ± 2.65 S.D.	21.31 ± 2.3 S.D.
Education (undergradute/postgraduate)	7/10	8/10	10/6
Handedness (r/l)	16/1	17/1	14/2

### Experimental design

The examination was performed in two separate sessions. At least two weeks of washout period was kept between the two occasions. During both sessions, all participants completed three levels of the n-back task before and after stimulation. Either the right or the left DLPFC was stimulated during the first examination, with the other hemisphere on the second occasion, respectively. The order of stimulation side was counterbalanced across participants. Three groups were formed: eighteen out of the fifty-one participants were given iTBS, seventeen of them received cTBS, and sixteen participants were assigned to the sham stimulation group. The experimental protocol was identical in each group, except for the type of stimulation.

### N-back task

**Figure 2:**
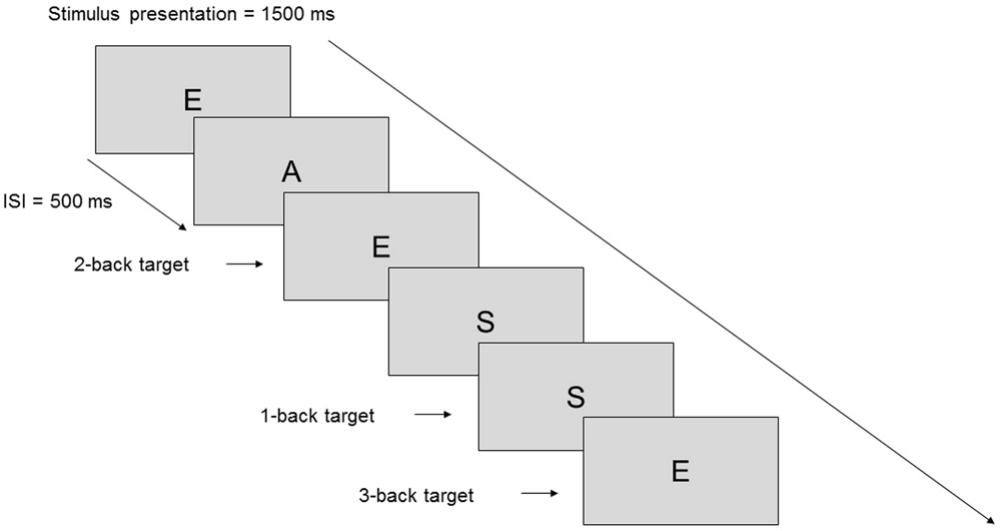
Illustration of the n-back task. Level 1-back, 2-back, and 3-back were tested separately.

The 1-back, 2-back, and 3-back version of the n-back task was administered consecutively^60^ using PsychoPy (version: v1.82.01)^61^. During the n-back tasks, random capital letter stimuli (A, C, E, I, K, L, S, O, R, T, U) were presented serially on the screen for 1500 ms with an interstimulus interval of 500 ms. Participants had to respond by pressing the space bar if the letter on the screen was the same as the letter presented one (1-back task), two (2-back task) or three (3-back task) trials earlier (Fig. 2). A total of 100 trials was completed at each level meaning a total of 300 trials per measurement. The frequency of target stimuli was set at 20% of all presented stimuli. The total duration of the tasks was about 15 minutes. We assumed that post-stimulation effects lasted during the entire examination, based on previous studies using TBS to modulate cognitive performance^22,25^. Reaction times (RT), number of hits, correct rejections, false alarms and misses were recorded.

### Theta-burst stimulation protocol

TBS was generated by a Magstim Rapid^2^ stimulator with a D70^2^ 70 mm figure-of-eight coil (The Magstim Company Ltd, Whitland, Wales, UK). Prior to the experiment, each participant in the iTBS and cTBS group went through an anatomical T1-weighted MRI-scan using a 1.5T GE Signa Excite HDxt scanner (Milwaukee, WI, USA): 3D IR-FSPGR: TR/TE/TI: 10.3/4.2/450 ms; flip angle: 15; ASSET: 2, FOC: 25 × 25 cm; matrix: 256 × 256; slice thickness: 1 mm. A TMS Neuronavigator (Brain Innovation, Maastricht, the Netherlands) with ultrasound CMS20 Measuring System (Zebris GmbH, Tübingen, Germany) was used to localize the target position on the scalp of each participant. Prior to this, 3D-brain models were created using participants’ MRI scans for accurate and individual targeting. Right and left DLPFC has been marked as the target area located on the 3D surface rendering of the brain, based on each participants’ gyral morphology (the anterior third of the middle frontal gyrus, Brodmann 9/46). We positioned the center of the coil over the target area, tangentially to the skull, with the handle pointing backward. During sham stimulation, the coil was rotated 45° away from the skull, with one wing of the coil being in contact with the scalp. Within the sham group, we administered the iTBS protocol for half of the participants, and cTBS pattern for the other half of the participants.

The cTBS and iTBS administration protocols in the current study was based on the original TBS stimulation pattern described by Huang *et al*.^5^. The cTBS pattern consisted of 3 pulses given at 50 Hz (gamma frequency) in every 200 ms (theta frequency intervals of 5 Hz) for 40 s. Thus, a total of 600 pulses of uninterrupted TBS was administered to each participant in the cTBS group. As for iTBS, a 2 s train was repeated every 10 s for 190 s in total. In one train, 3 pulses were given at 50 Hz, which resulted in 600 pulses for each subject in the iTBS group. The stimulation intensity was kept at 30% of the maximal stimulator output (MSO) of the Magstim Rapid^2^ stimulator, due to limitations in the maximal TBS intensity of the stimulator. We chose to administer equal intensity for all participants. This was based on evidence claiming that the individual adaptation of TMS intensity to the measured motor threshold does not necessarily lead to more prominent effects^62^. To make sure that the potential effects are not due to differences in the motor thresholds, we measured visible motor threshold (MT) without voluntary contraction prior to the two TBS sessions, at a separate occasion. It was defined as the lowest stimulation intensity applied over the right primary motor cortex (M1) required to elicit visible contraction of the left abductor pollicis brevis muscle in 3 out of 5 probes. If 80% of the individual MT did not reach 30% of MSO so that the intensity does not exceed the 80% of MT, the applied intensity was reduced by 20% of the MT. This occurred in one case in the cTBS group (27% of MSO) and in one case in the sham group (29% of MSO). The mean rMTs and ranges are given in Table 2. There was no difference between groups in terms of the mean rMT (F_(2,48)_ = 1.325, p = 0.275, n_p_^2^ = 0.52, BF_10_ = 0.385).

**Table 2:** rMT details.

	cTBS	iTBS	sham
Mean rMT (% of MSO)	62.35 ± 12.81 S.D.	59.72 ± 13 S.D.	55.69 ± 10.26 S.D.
Range (% of MSO)	34–78	43–80	36–70

### Statistical analysis

For statistical analysis with the frequentist approach, IBM SPSS Statistics 24.0 software package was used. N-back performance was evaluated by two scores: median reaction times (RT) and discriminability index (d′) used in the framework of signal detection theory^63^. We chose to use median RTs over mean scores to avoid the confounding effect of invalid (i.e. extremely short or extremely long) RTs^64^. For calculating d′ scores, we distinguished four types of answers: hits (correctly identified targets), misses (incorrectly identified targets as non-targets), false alarms (incorrectly identified non-targets as targets), and correct rejections (correctly identified non-targets). The d′ value is a highly sensitive statistical index involving both the ability to maximize hits and the efficiency of minimizing false alarms. It is calculated from the standard deviation of the signal and the noise distribution, with higher scores representing more readily detected signals (therefore greater discriminability)^65^. d′ scores were calculated individually as:

$$d^{\prime} =Z({\rm{hit}}\,{\rm{rate}})-Z({\rm{false}}\,{\rm{alarm}}\,{\rm{rate}}).$$

We analyzed RTs by a 3 × 2 × 2 × 2 mixed ANOVA with the cognitive load (LOAD: 1. 1-back; 2. 2-back; 3. 3-back), the time of administration (TIME: 1. pre-stimulation; 2. post-stimulation) and the side of the stimulation (SIDE: 1. right DLPFC; 2. left DLPFC) as within-subject factors, and stimulation type (GROUP: 1. iTBS; 2. cTBS; 3. sham) as between-subject factor. Considering that participants completed the 1-back task with an extremely high accuracy (99.53% in total), d′ scores were not calculated for this task. Thus, d′ scores have been analyzed by a 2 × 2 × 2 × 2 mixed ANOVA with the same parameters as for the RTs, excluding the 1-back condition. Pairwise comparisons of estimated marginal means (with Bonferroni correction for multiple testing) was used for follow-up on the significant main effects and interactions. Prior to analyzing TBS-induced effects, an additional mixed ANOVA with LOAD and SIDE as within-subject factors, and with GROUP as a between-subject factor was carried out, to test whether the three groups differed in terms of their pre-stimulation performance (factor levels were the same as previously defined).

In addition to the more conventional frequentist statistical approach, we also calculated Bayes-factors (BF) for the underpinning of our non-significant, but relevant results. Although the classical null-hypothesis testing mainly relies on the p-value, it is important to keep in mind that a non-significant result could mean at least two different things: (1) the null hypothesis is true; or (2) the collected data are not sensitive enough to distinguish between the null and an alternative hypothesis^66^. BF could be considered as the weight of evidence provided by the collected data, helping to differentiate between these two options^67^. Therefore, to compare the likelihood of our models favoring the H_0_ versus the H_1_ hypothesis, we performed Bayesian ANOVAs using JASP 0.8.03.01^68^ carried out by default prior. Here we report BF_10_ values that should be interpreted as follows: BF_10_ values between 1 and 0.33 indicate anecdotal, values between 0.33 and 0.1 substantial, and values below 0.1 strong evidence for H_0_. Reversely, values between 1 and 3 indicate anecdotal, values between 3 and 10 substantial, and values above 10 strong evidence in favor of H_1_^67^.

## Electronic supplementary material

Supplementary material
