# Supplementary material for: Continuous theta-burst stimulation over the dorsolateral prefrontal cortex inhibits improvement on a working memory task
Source: Sci Rep. 2018 Oct 4;8:14835. doi: 10.1038/s41598-018-33187-3 (PMC6172210; doi:10.1038/s41598-018-33187-3)
Supplement: Supplementary file 1 — Supplementary material [file 41598_2018_33187_MOESM1_ESM.pdf]

Supplementary Material

Continuous theta-burst stimulation over the dorsolateral prefrontal cortex inhibits improvement on a working memory task

Teodóra Vékony[a *], Viola Luca Németh[a], Adrienn Holczer[a], Krisztián Kocsis[a], Zsigmond Tamás Kincses[a], László Vécsei[a,b], Anita Must[b,c *]

[a] Department of Neurology, University of Szeged, Szeged, Hungary

[b] MTA-SZTE Neuroscience Research Group, Szeged, Hungary

[c] Institute of Psychology, University of Szeged, Szeged, Hungary

[*] Corresponding authors:

Anita Must MD PhD, Institute of Psychology, University of Szeged, Egyetem u. 2, H-6722, Szeged, Hungary, must.anita@med.u-szeged.hu

Teodóra Vékony, Department of Neurology, University of Szeged, Semmelweis u. 6, H-6725, Szeged, Hungary, vekony.teodora@med.u-szeged.hu

	DF	Error DF	F	p	n_p^2	BF_{10}
GROUP	2	48	2.993	.06	.111	.722
GROUP × SIDE	2	48	.471	.627	.019	.075
GROUP × LOAD	3.436	82.474	.537	.682	.019	.062
GROUP × SIDE × LOAD	3.925	94.190	.482	.627	.035	.086

Supplementary Table S1. Comparison of pre-stimulation median RTs between groups

	DF	Error DF	F	p	n_p^2	BF_{10}
GROUP	2	48	2.125	.131	.048	< .001
GROUP × SIDE	2	48	.586	.560	.024	.190
GROUP × LOAD	2	48	.065	.937	.003	.105
GROUP × SIDE × LOAD	2	48	.135	.375	.040	< .001

Supplementary Table S2. Comparison of pre-stimulation d’ scores between groups
